# Faricimab at 6 and 12 mg reduces pigment epithelium detachment in treatment-resistant macular neovascularization: an OCT and AI analysis

**DOI:** 10.1038/s41598-025-09910-2

**Published:** 2025-09-30

**Authors:** Ines D. Nagel, Melanie D. Tran, Haochen Zhang, Lingyun Cheng, Anna Heinke, Nehal N. Mehta, Dirk-Uwe Bartsch, Fritz G. P. Kalaw, Mohamed Morsy, Arthur J. Mueller, William R. Freeman

**Affiliations:** 1 Jacobs Retina Center, 9415 Campus Point Drive, La Jolla, CA 92093 USA; 2 Viterbi Family Department of Ophthalmology and Shiley Eye Institute, University of California, Berkeley, 92093 USA; 3 Division of Ophthalmology Informatics and Data Science, Viterbi Family Department of Ophthalmology and Shiley Eye Institute, University of California San Diego, La Jolla, CA 92093 USA; 4 Department of Ophthalmology, University Hospital Augsburg, 86156 Augsburg, Germany; 5 School of Medicine, University of California San Diego, San Diego, CA USA

**Keywords:** Choroidal neovascularization, Age-related macular degeneration, anti-VEGF resistance, Faricimab, Dose escalation, AI-based image analysis, Retinal diseases, Outcomes research

## Abstract

Macular neovascularization (MNV) in age-related macular degeneration (AMD) remains a therapeutic challenge, especially in eyes resistant to conventional anti-VEGF therapy. This study evaluates the anatomical and functional response to Faricimab in patients with persistent fluid despite intensified Aflibercept treatment and explores potential benefits of dose escalation. This cross-sectional study included 25 eyes from 23 patients with active MNV resistant to standard anti-VEGF therapy. All had persistent fluid on OCT despite monthly injections and received off-label double-volume Aflibercept 4 mg (0.1 ml) for at least three doses. Eyes with persistent fluid were switched to Faricimab 6 mg. In non-responders, Faricimab was further escalated to 12 mg (0.1 ml). Retinal fluid volumes and pigment epithelium detachment (PED) were analyzed using AI-based OCT segmentation. MNV activity was assessed using AI-based OCTA analysis. Faricimab 6 mg significantly reduced PED volume (*p* < 0.05), especially after the first two injections. However, changes in intraretinal and subretinal fluid were not significant. In the Faricimab 12 mg subgroup, no additional anatomical benefit was observed. OCTA showed a trend toward reduced vascular activity after switching to Faricimab 6 mg, but no further change with dose escalation. In MNV eyes resistant to high-dose Aflibercept, Faricimab 6 mg provides meaningful anatomical improvement, particularly in PED volume. However, escalating Faricimab to 12 mg offers no additional benefit, suggesting therapeutic saturation at the standard dose.

## Introduction

Age-related macular degeneration (AMD) with macular neovascularization (MNV) presents a clinical challenge, particularly in patients who exhibit resistance to conventional intravitreal treatment regimens^1^. Despite adhering to standard 4-week treatment schedules with anti-VEGF-agents, a subset of patients continues to demonstrate persistent subretinal and intraretinal fluid, suggesting suboptimal response^2^. In such resistant cases, clinical management often necessitates individualized strategies^3^. This might include dose escalation or switching medications^4–8^. Given the lack of standardized approaches for these resistant cases, treatment regimens tend to vary between institutions and providers. In our practice, we have implemented a regimen aimed at improving clinical outcomes for these difficult-to-treat patients.

Aflibercept’s non-inferiority to Ranibizumab has already been introduced by effectively treating neovascular age-related macular degeneration^9,10^. It functions as a “fusion protein” that mimics a vascular endothelial growth factor (VEGF) receptor. It selectively binds to VEGF-A, VEGF-B and placental growth factor which are essential for angiogenesis. By binding to these growth factors, a formation of new pathological blood vessels is prevented. Moreover, the effect of VEGF to increase vascular permeability, is prevented^11^.

Faricimab is a bispecific monoclonal antibody designed for use in retinal diseases. Its non-inferiority to Aflibercept has been observed by treating neovascular age-related macular degeneration^12,13^. Faricimab targets two pathways involved in disease pathogenesis: the VEGF-A pathway and the angiopoietin-2 (Ang-2) pathway. As Ang-2 expression increases, the protective Ang-1/Tie-2 loses its ability to maintain vascular integrity. By inhibiting Ang-2, blood vessel stabilization, endothelial cell junction and reduction of vascular permeability is ensured^14^.

In the past, it has been demonstrated that, in cases of resistant MNVs, an increased dose can improve the therapeutic response to medication^15^. This concept has been clinically implemented with the introduction of Eylea HD (8 mg Aflibercept), which is approved for patients with neovascular AMD (nAMD) and has shown improved durability and efficacy in certain cases^16^.

Faricimab is currently approved at a 6 mg dose for nAMD. In our study, we selected this as the baseline, on-label treatment dose. For patients with an incomplete response to Faricimab 6 mg, we investigated an escalation to 12 mg. Although this higher dose is not currently approved and there is no published data on Faricimab 12 mg so far, the rationale for dose escalation that higher-dose anti-VEGF strategies were explored with other agents, suggesting potential benefit in cases of suboptimal response^15^.

In this study, we aimed to investigate whether Faricimab 6 mg leads to a superior anatomical and functional outcome to Aflibercept 4 mg in patients with resistant MNV. Additionally, we aim to assess whether an escalation to off-label Faricimab 12 mg provides further benefit in cases with an incomplete response to Faricimab 6 mg.

## Methods

This cross-sectional study included patients with active macular neovascularization (MNV) who were undergoing intravitreal injections at a single ophthalmology center.

The study was approved by the Institutional Review Board at the University of California–San Diego, California, USA (IRB # 120516). This study complied with the Health Insurance Portability and Accountability Act of 1996 and patient informed consent was obtained by the institution’s protocol. Data was anonymized for patient safety and all collection and analysis was conducted by the Principles of the Declaration of Helsinki.

A total of 25 eyes from 23 patients met the inclusion criteria and were analyzed. Thirty patients were excluded due to one of more of the following: (1) failure to receive the specific treatment regimen under investigation, (2) poor image quality of Optical Coherence Tomography (OCT) scans that precluded analysis (signal to noise ratio below 20 dB or presence of significant motion artifacts that prevented accurate retinal layer assessment), or (3) classification as Type 3 MNV or polypoidal lesions, which were not within the study’s scope.

Initially, all patients received monthly intravitreal injections of conventional anti-vascular endothelial growth factor (anti-VEGF) therapy. For cases exhibiting persistent intraretinal or subretinal fluid on OCT despite regular dosing, treatment was escalated to an off-label, double-volume dose of Aflibercept (4 mg in 0.1 ml, Regeneron Pharmaceuticals Inc., New York, USA). This intensified treatment was administered for a minimum of three consecutive injections. Persistent fluid was defined as continued fluid presence on OCT following multiple standard-dose injections, administered on a fixed schedule.

The total duration of anti-VEGF therapy was recorded for each patient. Following inadequate response to Aflibercept 4 mg (defined as persistent fluid after at least three injections), treatment was transitioned to the approved dose of Faricimab (6 mg in 0.05 ml, Genentech Inc., San Francisco, USA). Baseline OCT scans were obtained after the final Aflibercept injection and served as the reference for further assessment.

Baseline scans were evaluated to classify the MNV subtype based on established criteria into Type 1, Type 2, or Type 3 lesions^17,18^. OCT scans were subsequently analyzed at multiple time points: after each of the first three injections of Faricimab 6 mg and following the final injection in the treatment sequence. In addition, OCT Angiography (OCTA) was used to provide detailed visualization of neovascular structures and assess treatment response over time. Best-corrected visual acuity (BCVA) was measured at all corresponding time points.

Quantitative analysis of OCT volume scans was performed using RetinAI software (RetinAI Medical AG, Bern, Switzerland). This software enabled precise segmentation and measurement of retinal thickness (from the internal limiting membrane [ILM] to Bruch’s membrane [BM]), as well as the volume of intraretinal and subretinal fluid, based on the Early Treatment Diabetic Retinopathy Study (ETDRS) grid^19^. Pigment epithelial detachment (PED) volume was also quantified. To ensure accuracy, all automated segmentations were reviewed by a retina specialist (IN). In case of visible mismatch, manual correction was performed using the integrated editing tools in every scan.

For statistical analysis, fluid volumes within the 3 mm inner ring of the ETDRS grid (representing the central macular zone) were selected to monitor changes over time. PED volume measurements were similarly restricted to this region.

Additionally, any history of vitrectomy was recorded, along with the clinical indications for the procedure. Changes in retinal fluid and PED volume over time were statistically analyzed to evaluate the therapeutic effectiveness of Faricimab.

A subgroup of patients who showed no significant anatomical response to both Aflibercept and at least three injections of Faricimab 6 mg received an escalated dose of Faricimab (12 mg in 0.1 ml). For this group, baseline measurements were taken after the last 6 mg Faricimab injection. Further OCT analyses were conducted following the first, second, and third injections of the 12 mg Faricimab dose, with an additional follow-up assessment conducted at least three months after the third injection. During this follow-up period, the treatment interval was adjusted based on clinical evaluation of fluid recurrence or persistence. OCT segmentation and data collection for this subgroup followed the same methodology as described for the initial treatment cohort.

To assess vascular activity, OCTA images were analyzed using an AI tool previously trained by our team to evaluate MNV activity. The AI tool classified MNV status as follows: 0 = active, 1 = remission, 2 = dry, 3 = normal. To ensure accuracy, all automated segmentations were reviewed by a retina specialist (IN). In case of visible mismatch, manual correction was performed using the integrated editing tools in every scan^20^.

**Fig. 1:**
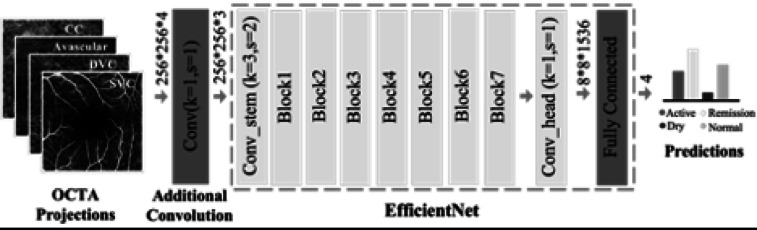
Modified EfficientNet-B5 on four-channel OCTA images for AMD stage classification using data augmentation, oversampling, and ensemble training

Due to the limited availability of longitudinal data for fine-tuning a new classification model, we adopted the AMD stage grading model proposed in our previous work^21^. Specifically, we employed EfficientNet-B5^22^ as the backbone network, incorporating necessary modifications to accommodate the unique characteristics of OCTA data (Fig. 1). EfficientNet-B5 is designed to process three-channel RGB inputs typical of natural images, whereas our OCTA dataset consists of four projection images at varying depths. To address this discrepancy, we introduced an additional convolutional layer with a kernel size of one to process the four-channel input appropriately. Furthermore, we adjusted the final fully connected layer to output four categories—active, remission, dry, and normal—corresponding to our classification task.

To initialize the model, we pre-trained the network on ImageNet^23^ a large-scale dataset for natural image classification, before fine-tuning it on our specific OCTA projection images, which included a mixture of Heidelberg and Optovue samples. We adhered to a standard training-validation-testing split: the Heidelberg Spectralis dataset (1,478 samples) was partitioned into 1,102 for training, 276 for validation, and 100 for testing, while the Optovue Solix dataset (1,003 samples) was split into 754 for training, 189 for validation, and 60 for testing. Prior to training, each OCTA projection was downsampled from 512 × 512 to 256 × 256 pixels. Data augmentation techniques were applied to enhance dataset diversity, including random flipping, rotation, cropping with resizing, gamma transformation, Gaussian smoothing to account for intensity variations, and grid distortion for shape augmentation. Additionally, sample-wise normalization was applied to standardize intensity distributions.

The deep learning models were implemented using the PyTorch framework. Training was conducted using the Adam optimizer with a weight decay of 0.00001. The initial learning rate was set to 0.001 and progressively reduced by a factor of 10 after 400, 800, and 1,200 epochs over a total of 1500 training epochs. We employed a cosine loss function which has demonstrated effectiveness in small-scale datasets^24^. To mitigate class imbalance, oversampling techniques were applied to underrepresented categories. For model evaluation, we performed a five-fold cross-validation, yielding five independent classifiers. The final predictions were obtained through an ensemble of these models to improve robustness and generalizability^21–24^.

### Statistical analysis

The retinal fluid volume, including intraretinal and subretinal fluid, before switching to single dose Faricimab was compared in pairs with subsequent follow-ups after each Faricimab loading. For the fluid volume comparison, Wilcoxon Signed Rank test was used. Similarly, the volume of PED was also assessed in pairs to compare the volume before Faricimab loading and the subsequent volume at the follow-ups. For the volume of PED comparison, paired t-test was used. For the patients who failed to respond to single dose Faricimab, the retinal fluid and PED volume before double dose of Faricimab were used as a baseline to compare with the volumes at subsequent follow-ups after the double dose in a similar statistical evaluation. All the tests were two-tailed and performed within JUMPER statistical software, version pro 18. Type I error of 5% was allowed to judge the statistical significance.

## Results

A total of 25 eyes from 23 patients met the inclusion criteria and were included in the final analysis. All eyes demonstrated treatment resistance, characterized by persistent intraretinal and/or subretinal fluid despite receiving Aflibercept at the standard dosage of 2 mg. Prior to this regimen, patients had undergone intravitreal anti-VEGF therapy using a treat-and-extend protocol, with a mean treatment duration of 1472.96 days (SD ± 1073.19).

All eyes received at least three four-weekly injections of Aflibercept 4 mg before being transitioned to Faricimab 6 mg. The mean age of the cohort was 81.6 years (SD ± 5.8), 56% female patients were included. Only one of the included patients was phakic (4%), she remained phakic throughout the whole study period. A total of 17 PEDs (68%) were classified as fibrovascular, while the remaining 8 PEDs (32%) exhibited a multilayered morphology. There was no significant diference in PED morphology between the individual groups (*p* > 0.05).

The distribution of MNV subtypes included Type 1 MNV in 72% eyes and Type 2 MNV in 28%. Six eyes (22%) had received a vitrectomy in the past (four eyes underwent epiretinal membrane peel, one eye underwent vitrectomy for vitreous heme and one eye underwent a retinal detachment repair).

### Treatment response to Faricimab 6 mg

**Fig. 2:**
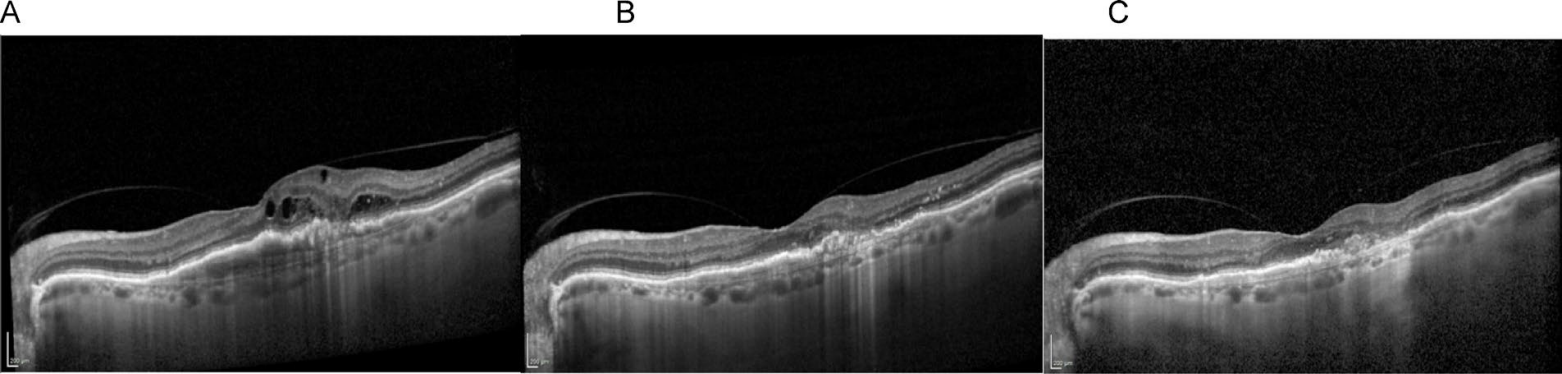
Disease progression over time – (**A**) Baseline, (**B**) Faricimab 6 mg at 8 weeks, and (**C**) Follow-up at 18 months with treat-and-extend Faricimab 6 mg regimen. Reduction in intraretinal fluid and PED volume can be observed.

Baseline was defined as the final visit following Aflibercept 4 mg treatment. Starting from this time point, an overall significant reduction in PED volume was observed after the first, second, and third injections of Faricimab 6 mg (*p* = 0.0072, *p* < 0.0001, and *p* = 0.0051, respectively (Table 1; Fig. 2). This reduction remained statistically significant during the follow-up period (*p* = 0.0305).

**Fig. 3:**
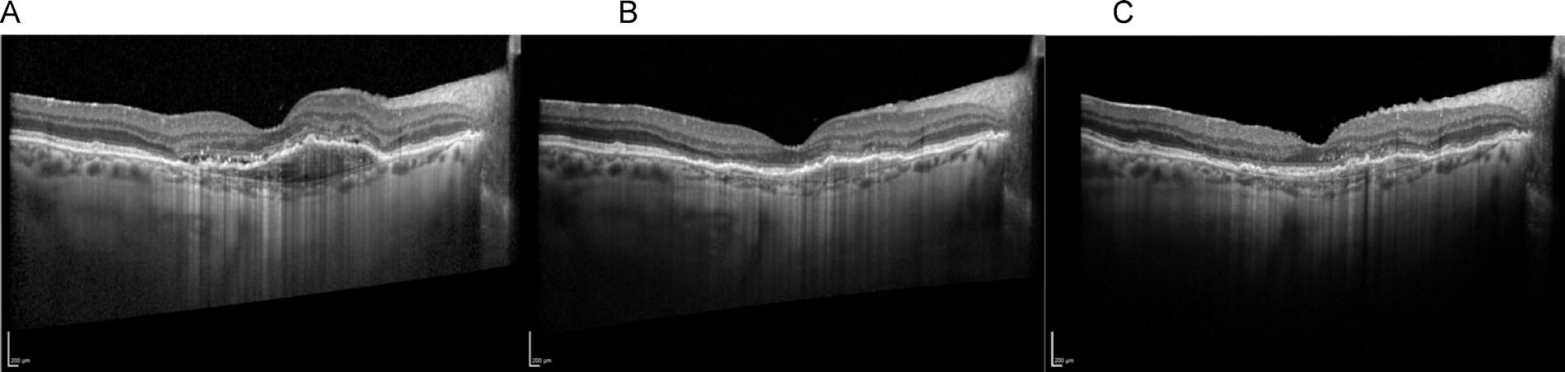
Disease progression over time – (**A**) Baseline, (**B**) Faricimab 6 mg at 8 weeks, and (**C**) Follow-up at 24 months with treat-and-extend Faricimab 6 mg regimen. Reduction in subretinal fluid and PED volume can be observed

**Fig. 4:**
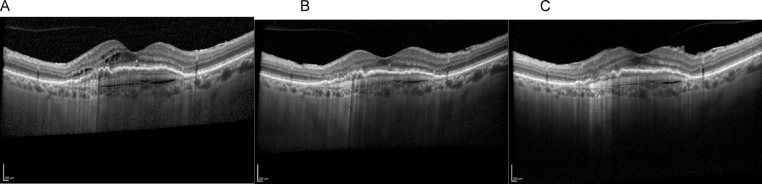
Disease progression over time – (**A**) Baseline, (**B**) Faricimab 6 mg at 8 weeks, and (**C**) Follow-up at 18 months with treat-and-extend Faricimab 6 mg regimen. Reduction in intraretinal fluid and PED volume can be observed

Notably, the greatest decrease in PED volume occurred following the first and second injection (Figs. 2, 3 and 4) Patients demonstrating particularly favorable anatomical response by this point were transitioned to extended treatment intervals based on clinical judgment, rather than a standardized protocol. This reflects a drug regimen tailored to individual response.

In contrast, no statistically significant changes were detected in intraretinal or subretinal fluid volumes at any time point (*p* > 0.05). Although a numerical reduction was observed, it did not reach statistical significance across the first three injections or the follow-up period.

Similarly, BCVA did show a trend towards improving visual acuity but without statistical significance.

**Table 1:** Pigment epithelium detachment (PED) volume in nanoliters, compared from baseline to faricimab 6 mg after 4, 8, 12 weeks and the follow up period. PED volume was statistical significantly reduced at all timepoints.

PED Volume Faricimab 6 mg Overall (nL)	Baseline (Eylea 4 mg)	First Injection (Faricimab 6 mg)	Second Injection (Faricmab 6 mg)	Third Injection (Faricimab 6 mg)	Follow Up (Faricimab 6 mg)	*p*-value
Mean	309.80769	240.84	253.68	276.36	266.23077	*p* < 0.05
Std Dev	342.10244	268.56527	286.65117	312.95485	292.03442	
Std Err Mean	67.091809	53.713055	57.330234	62.590971	57.272662	
Upper 95% Mean	447.98586	351.6983	372.00379	405.54141	384.18603	
Lower 95% Mean	171.62953	129.9817	135.35621	147.17859	148.27551	

### Response to escalated Faricimab 12 mg in non-responders

A subset of 10 eyes that failed to respond adequately to three or more injections of Faricimab 6 mg – defined by persistent or increasing fluid – were escalated to a higher dose of Faricimab (12 mg in 0.1 ml). This subgroup had a mean age of 80.5 years (SD ± 4.5), with Type 1 MNV in 55% and Type 2 MNV in 45% of cases. Four eyes (40%) underwent vitrectomy in the past.

**Fig. 5:**
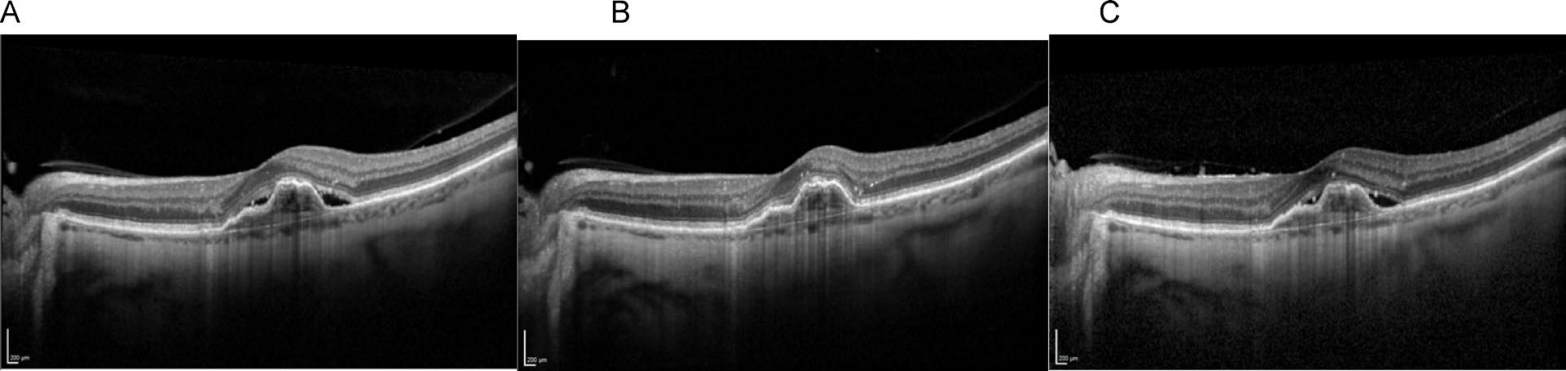
Disease progression over time – (**A**) Baseline, (**B**) Faricimab 6 mg at 8 weeks, and (**C**) Follow-up at 6 months, switched to monthly Faricimab 12 mg regimen. Recurrence of subretinal fluid and no change in PED size can be observed

No statistically significant anatomical response was observed in this group. Neither intraretinal nor subretinal fluid volumes, nor PED volumes, showed significant change following the administration of Faricimab 12 mg (*p* > 0.05). Although a continued reduction in PED size was noted when compared to baseline, this effect did not reach statistical significance (Fig. 5).

**Table 2:** Fluid volume in nanoliters, compared from baseline to faricimab 12 mg after 4, 8, 12 weeks and the follow up period. Fluid volume was not statistical significantly reduced at any timepoint.

Fluid Volume Faricimab 12 mg Overall (nL)	Baseline (Faricimab 6 mg)	First Injection (Faricimab 12 mg)	Second Injection (Faricmab 12 mg)	Third Injection (Faricimab 12 mg)	Follow Up (Faricimab 12 mg)	*p*-value
Mean	114.14286	86.214286	104.07692	78.727273	105.64286	*p* > 0.05
Std Dev	174.62966	162.23685	141.38804	173.49991	186.81526	
Std Err Mean	46.671739	43.359623	39.213986	52.312191	49.928477	
Upper 95% Mean	214.97102	179.88706	189.51686	195.2861	213.50677	
Lower 95% Mean	13.314696	7.458485	18.636988	− 37.83155	− 2.22106	

### Subgroup analysis based on response to 6 mg Faricimab

Due to the differential response to Faricimab 6 mg, we reanalyzed the eyes separately. All eyes that received Faricimab 6 mg were divided into two groups: Group 1 (eyes that subsequently responded to Faricimab 6 mg) and Group 2 (eyes that were later classified as non-responders to Faricimab 6 mg). Table 2 presents the mean values for each group. At baseline, there was no significant difference between the groups in the amount of intraretinal and subretinal fluid (*p* = 0.53). Interestingly, however, a comparison of the two groups over the course of treatment revealed that Group 1 showed a significant response to Faricimab 6 mg, whereas Group 2 did not exhibit a significant reduction in fluid at any time point (Table 3).

**Table 3:** Comparison of mean fluid volumes between group 1 and group 2 across timepoints following treatments with faricimab 6 mg.

Intraretinal and subretinal Fluid (nL)	Baseline	*p*-value (Group 1 vs. Group 2)	First Dose 6 mg	*p*-value (compared to baseline)	Second Dose 6 mg	*p*-value (compared to baseline)	Third Dose 6 mg	*p*-value (compared to baseline)	Follow Up 6 mg	*p*-value (compared to baseline)
Mean Group 1	50.83	*p* = 0.53	8.0	*p* < 0.0001	2.08	*p* < 0.0001	8.83	*p* < 0.0001	6.55	*p* = 0.027
Std Dev Group 1	56.52		27.09		4.54		16.49		16.67	
Mean Group 2	66.50		127.69	*p* = 0.37	65.38	*p* = 0.98	145.54	*p* = 0.25	114.14	*p* = 0.35
Std Dev Group 2	63.70		228.57		131.19		229.26		174.63	

### OCTA-based vascular activity

An OCTA-based evaluation of MNV activity revealed a trend toward reduced vascular activity in eyes switched from Aflibercept to Faricimab 6 mg, with a higher proportion of MNV lesions categorized as inactive at follow-up. However, this trend was not statistically significant (*p* = 0.25), with 30% with an inactive mature vessel presence at baseline and 44% with inactive vessels after treatment. No such trend was observed in eyes escalated to Faricimab 12 mg.

## Discussion

The principal finding of this study is that Faricimab, administered at the standard 6 mg dose, demonstrated a significant reduction in PED volume in eyes with MNV resistant to Aflibercept, particularly during the initial loading phase with monthly injections. Importantly, this anatomical improvement persisted at follow-up, indicating a sustained treatment effect. While trends toward a reduction in intraretinal and subretinal fluid, as well as improvements in BCVA were observed, these changes did not reach statistical significance.

Notably, approximately half of the cases exhibited a dramatic, statistically significant response, whereas the other half appeared to be treatment-resistant, highlighting the heterogeneity in therapeutic effect across the cohort. Furthermore, administration of a 12 mg dose did not give any additional benefit for those non-responders.

In the management of treatment-resistant MNV, the primary therapeutic objective remains the resolution of intraretinal and subretinal fluid, as fluid accumulation is a known contributor to vision loss^25^. Intravitreal anti-VEGF agents, including Faricimab, are commonly employed to achieve this goal^26^. Although PED is not typically the main target in treatment algorithms^27^ the consistent reduction observed in our cohort suggests potential clinical relevance, especially in cases where PED contributes to retinal instability. Interestingly, half of the patients who were resistant to Eylea 4 mg responded well to Faricimab, indicating a possible advantage of the dual-mechanism agent.

Our findings suggest that Faricimab may exert a more pronounced effect on PED than on retinal fluid, at least in our selected cohort. This may reflect the dual mechanisms of action of Faricimab, which inhibits both VEGF-A and Angiopoietin-2 (Ang-2). This broader mechanism, compared to VEGF-A monotherapy, may contribute to vascular stabilization and reduced leakage, particularly in fibrovascular PEDs characterized by neovascular tissues, fibrovascular components, and exudation beneath the RPE. In this context, the observed PED reduction could represent a secondary marker of disease control.

In cases where fluid levels remained unchanged despite Faricimab 6 mg therapy, a higher degree of treatment resistance is possible. Prior evidence has shown that increasing the dosage of Aflibercept enhances its efficacy, presumably through higher VEGF-A saturation^15^. However, the dual-mechanism of Faricimab, may achieve near-complete target saturation at the standard 6 mg dose. Consequently, additional benefit from dose escalation to 12 mg might be limited, particularly if receptor occupancy is already maximized.

Nevertheless, a recently published study involving 25 eyes previously requiring bimonthly aflibercept injections, 56% showed a reduction or complete resolution of exudative fluid two months after switching to Faricimab, despite no significant change in central retinal thickness or visual acuity^26^. In another study evaluating a similar cohort, switching to Faricimab was found to be effective with a reduction in intraretinal fluid but only marginal improvements in BCVA^4^. While the overall anatomical response was variable, these results align with our own observation that Faricimab may achieve disease control in a subset of refractory patients.

Due to the differential response to Faricimab 6 mg in our cohort, we reanalyzed the treated eyes by dividing them into the responder group and the non-responders, that were switched to Faricimab 12 mg eventually. Longitudinal comparison revealed a clear divergence with no anatomical improvement at any time point for the non-responders. These findings highlight the importance of early identification of non-responders to guide treatment adjustment.

This study has several limitations. The relatively small sample size, particularly in the subgroup treated with Faricimab 12 mg, reduces the statistical power of subgroup analyses. Furthermore, several patients had previously undergone vitrectomy, a factor that may influence intraocular pharmacokinetics and drug efficacy. While preclinical data suggest that vitrectomy does not significantly alter the clearance of intravitreal drugs^28,29^ the overrepresentation of vitrectomized eyes in the 12 mg cohort introduces a potential confounder that warrants further investigation.

Another limitation relates to the individualized, response-based treatment regimen employed in this study. Patients demonstrating favorable anatomical response after two Faricimab injections were shifted to extended intervals, deviating from a fixed dosing protocol. While this approach reflects real-world clinical practice, it introduces variability that may have affected outcome interpretation. Notably, in some cases, PED reaccumulated following interval extension. Had all patients continued a strict monthly regimen for three months, the degree of fluid or PED reduction may have differed, potentially improving both anatomical and functional outcomes.

To our knowledge, this is the first study to evaluate a stepwise escalation strategy in MNV patients resistant to both standard and high-dose Aflibercept, using increased doses of Faricimab. These findings offer clinically relevant insights into treatment individualization, especially in settings where access to alternative medications is limited by regulatory or reimbursement constraints.

It is important to underscore that this study does not propose PED as a primary therapeutic agent in MNV management. Rather, our findings suggest that PED can serve as an adjunctive maker of disease activity and treatment response, particularly in fibrovascular subtypes. The reduction in PED observed with Faricimab treatment reinforce its potential role in stabilizing the retinal structure, even when resolution of fluid is incomplete.

## Data Availability

The anonymized dataset used and analyzed during the current study is available from the corresponding author on reasonable request.

## References

[1] Arcinue, C. A. et al. One-year outcomes of aflibercept in recurrent or persistent neovascular age-related macular degeneration. *Am J Ophthalmol* 159, 426–436.e422 (2015). 10.1016/j.ajo.2014.11.022 10.1016/j.ajo.2014.11.022 PMC4329256 25461263

[2] Bakall, B. et al. Aflibercept therapy for exudative age-related macular degeneration resistant to bevacizumab and ranibizumab. *Am J Ophthalmol* 156, 15–22.e11 (2013). 10.1016/j.ajo.2013.02.017 10.1016/j.ajo.2013.02.017 23706500

[3] Mettu, P. S., Allingham, M. J. & Cousins, S. W. Incomplete response to Anti-VEGF therapy in neovascular AMD: exploring disease mechanisms and therapeutic opportunities. *Prog Retin Eye Res.* **82**, 100906. 10.1016/j.preteyeres.2020.100906 (2021). 33022379 10.1016/j.preteyeres.2020.100906 PMC10368393

[4] Schneider, M. et al. Short-term outcomes of treatment switch to faricimab in patients with aflibercept-resistant neovascular age-related macular degeneration. *Graefes Arch. Clin. Exp. Ophthalmol.* **262**, 2153–2162. 10.1007/s00417-024-06421-0 (2024). 38416237 10.1007/s00417-024-06421-0 PMC11222265

[5] Chang, A. A. et al. Intravitreal Aflibercept for treatment-resistant neovascular age-related macular degeneration. *Ophthalmology* **121**, 188–192. 10.1016/j.ophtha.2013.08.035 (2014). 24144450 10.1016/j.ophtha.2013.08.035

[6] Broadhead, G. K., Keenan, T. D. L., Chew, E. Y., Wiley, H. E. & Cukras, C. A. Comparison of agents using higher dose anti-VEGF therapy for treatment-resistant neovascular age-related macular degeneration. *Graefes Arch. Clin. Exp. Ophthalmol.* **260**, 2239–2247. 10.1007/s00417-021-05547-9 (2022). 35092447 10.1007/s00417-021-05547-9

[7] Ashraf, M., Banaee, T., Silva, F. Q. & Singh, R. P. Switching Anti-Vascular endothelial growth factors in refractory neovascular Age-Related macular degeneration. *Ophthalmic Surg. Lasers Imaging Retina*. **49**, 166–170. 10.3928/23258160-20180221-03 (2018). 29554383 10.3928/23258160-20180221-03

[8] Abri Aghdam, K., Seidensticker, F., Pielen, A., Framme, C. & Junker, B. The short-term effects of Aflibercept on the size of choroidal neovascularization lesion in treatment-resistant neovascular age-related macular degeneration as determined by spectral-domain optical coherence tomography. *Lasers Surg. Med.* **48**, 668–677. 10.1002/lsm.22531 (2016). 27111455 10.1002/lsm.22531

[9] Heier, J. S. et al. Intravitreal Aflibercept (VEGF trap-eye) in wet age-related macular degeneration. *Ophthalmology* **119**, 2537–2548. 10.1016/j.ophtha.2012.09.006 (2012). 23084240 10.1016/j.ophtha.2012.09.006

[10] Kaiser, P. K. et al. Long-term safety and visual outcome of intravitreal Aflibercept in neovascular Age-Related macular degeneration: VIEW 1 extension study. *Ophthalmol. Retina*. **1**, 304–313. 10.1016/j.oret.2017.01.004 (2017). 31047516 10.1016/j.oret.2017.01.004

[11] Singh, S. R., Dogra, A., Stewart, M., Das, T. & Chhablani, J. Intravitreal Ziv-Aflibercept: clinical effects and economic impact. *Asia Pac. J. Ophthalmol. (Phila)*. **6**, 561–568. 10.22608/apo.2017263 (2017). 28971631 10.22608/APO.2017263

[12] Heier, J. S. et al. Efficacy, durability, and safety of intravitreal faricimab up to every 16 weeks for neovascular age-related macular degeneration (TENAYA and LUCERNE): two randomised, double-masked, phase 3, non-inferiority trials. *Lancet* **399**, 729–740. 10.1016/s0140-6736(22)00010-1 (2022). 35085502 10.1016/S0140-6736(22)00010-1

[13] Khanani, A. M. et al. TENAYA and LUCERNE: Two-Year results from the phase 3 neovascular Age-Related macular degeneration trials of faricimab with Treat-and-Extend dosing in year 2. *Ophthalmology* **131**, 914–926. 10.1016/j.ophtha.2024.02.014 (2024). 38382813 10.1016/j.ophtha.2024.02.014

[14] Penha, F. M. et al. Review of real-world evidence of dual Inhibition of VEGF-A and ANG-2 with faricimab in NAMD and DME. *Int. J. Retina Vitreous*. **10**, 5. 10.1186/s40942-024-00525-9 (2024). 38233896 10.1186/s40942-024-00525-9 PMC10795384

[15] You, Q. S. et al. High-dose high-frequency aflibercept for recalcitrant. *Retina* **38**, 1156–1165 10.1097/iae.0000000000001726 (2018). 28604541 10.1097/IAE.0000000000001726 PMC5726954

[16] Lanzetta, P. et al. Intravitreal Aflibercept 8 mg in neovascular age-related macular degeneration (PULSAR): 48-week results from a randomised, double-masked, non-inferiority, phase 3 trial. *Lancet* **403**, 1141–1152. 10.1016/s0140-6736(24)00063-1 (2024). 38461841 10.1016/S0140-6736(24)00063-1

[17] Jung, J. J. et al. The incidence of neovascular subtypes in newly diagnosed neovascular age-related macular degeneration. *Am J Ophthalmol* 158, 769–779.e762 (2014). 10.1016/j.ajo.2014.07.006 10.1016/j.ajo.2014.07.006 25034111

[18] Yannuzzi, L. A. et al. Retinal angiomatous proliferation in age-related macular degeneration. *Retina* **21**, 416–434. 10.1097/00006982-200110000-00003 (2001). 11642370 10.1097/00006982-200110000-00003

[19] Grading diabetic retinopathy. From stereoscopic color fundus photographs–an extension of the modified airlie house classification. ETDRS report number 10. Early treatment diabetic retinopathy study research group. *Ophthalmology* **98**, 786–806 (1991). 2062513

[20] Deussen, D. N. et al. Effect of manual OCTA segmentation correction to improve image quality and visibility of choroidal neovascularization in AMD. *Sci. Rep.* **14**, 13990. 10.1038/s41598-024-61551-z (2024). 38886462 10.1038/s41598-024-61551-z PMC11183238

[21] Heinke, A. et al. Cross-instrument optical coherence tomography-angiography (OCTA)-based prediction of age-related macular degeneration (AMD) disease activity using artificial intelligence. *Sci. Rep.* **14**, 27085. 10.1038/s41598-024-78327-0 (2024). 39511248 10.1038/s41598-024-78327-0 PMC11544254

[22] Deng, J. et al. ImageNet: A large-scale hierarchical image database. *2009 IEEE Conference on Computer Vision and Pattern Recognition*, 248–255 (2009).

[23] Tan, M. & Le, Q. V. EfficientNet: rethinking model scaling for convolutional neural networks. *ArXiv* (2019). abs/1905.11946.

[24] Barz, B. & Denzler, J. Deep Learning on Small Datasets without Pre-Training using Cosine Loss. *IEEE Winter Conference on Applications of Computer Vision (WACV)*, 1360–1369 (2019)., 1360–1369 (2019). (2020).

[25] Schmidt-Erfurth, U., Vogl, W. D., Jampol, L. M. & Bogunović, H. Application of automated quantification of fluid volumes to Anti-VEGF therapy of neovascular Age-Related macular degeneration. *Ophthalmology* **127**, 1211–1219. 10.1016/j.ophtha.2020.03.010 (2020). 32327254 10.1016/j.ophtha.2020.03.010

[26] Tamiya, R. et al. Therapeutic effects of faricimab on aflibercept-refractory age-related macular degeneration. *Sci. Rep.* **13**, 21128. 10.1038/s41598-023-48190-6 (2023). 38036627 10.1038/s41598-023-48190-6 PMC10689783

[27] de Massougnes, S., Dirani, A., Mantel, I., Good visual outcome & at 1 year in neovascular age-related macular degeneration with pigment epithelium detachment. Factors influencing the treatment response. *Retina* **38**, 717–724. 10.1097/iae.0000000000001613 (2018). 28368974 10.1097/IAE.0000000000001613

[28] Ahn, J. et al. Pharmacokinetics of intravitreally injected bevacizumab in vitrectomized eyes. *J. Ocul Pharmacol. Ther.* **29**, 612–618. 10.1089/jop.2013.0009 (2013). 23735192 10.1089/jop.2013.0009 PMC3757534

[29] Ahn, S. J. et al. Intraocular pharmacokinetics of Ranibizumab in vitrectomized versus nonvitrectomized eyes. *Invest. Ophthalmol. Vis. Sci.* **55**, 567–573. 10.1167/iovs.13-13054 (2014). 24398088 10.1167/iovs.13-13054

